# Intestinal Anti-Endomysium Antibodies Are a Useful Tool for Diagnosing Celiac Disease in Pediatric and Adult Patients

**DOI:** 10.3390/nu16172979

**Published:** 2024-09-04

**Authors:** Chiara Zanchi, Fabiana Ziberna, Alessia Padoin, Alessia Visintin, Fabio Monica, Catrin Simeth, Renato Cannizzaro, Paola Pelizzo, Anna Maria Baragiotta, Piero Brosolo, Josefina Panos Zamora, Maurizio Zilli, Giorgia Fontana, Grazia Di Leo, Sara Lega, Matteo Bramuzzo, Luca Ronfani, Luigina De Leo, Tarcisio Not

**Affiliations:** 1 Institute for Maternal and Child Health, I.R.C.C.S. Burlo Garofolo, Via dell’Istria 65/1, 34100 Trieste, Italy; chiara.zanchi@burlo.trieste.it (C.Z.); fabiana.ziberna@burlo.trieste.it (F.Z.); giorgia.fontana@burlo.trieste.it (G.F.); grazia.dileo@burlo.trieste.it (G.D.L.); sara.lega@burlo.trieste.it (S.L.); matteo.bramuzzo@burlo.trieste.it (M.B.); luca.ronfani@burlo.trieste.it (L.R.); tarcisio.not@burlo.trieste.it (T.N.); 2 Gastroenterology and Digestive Endoscopy Unit, Academic Hospital Cattinara, Strada di Fiume, 447, 34149 Trieste, Italy; alessiap97@gmail.com (A.P.); alessia.visintin@asugi.sanita.fvg.it (A.V.); fabio.monica@asugi.sanita.fvg.it (F.M.); catrin.simeth@asugi.sanita.fvg.it (C.S.); 3 Unit of Oncological Gastroenterology, Centro di Riferimento Oncologico di Aviano (CRO) I.R.C.C.S., Via Franco Gallini, 2, 33081 Aviano, Italy; rcannizzaro@cro.it (R.C.); paola.pelizzo@cro.it (P.P.); 4 Department of Medical, Surgical and Health Sciences, University of Trieste, Piazzale Europa, 1, 34127 Trieste, Italy; 5 Gastroenterology Unit, Santa Maria degli Angeli Pordenone General Hospital, Via Montereale, 24, 33170 Pordenone, Italy; annamaria.baragiotta@asfo.sanita.fvg.it (A.M.B.); piero.brosolo@asfo.sanita.fvg.it (P.B.); 6 Gastroenterology and GI Endoscopy Unit, University Hospital of Udine, Piazzale Santa Maria della Misericordia, 15, 33100 Udine, Italy; josefina.panos@asufc.sanita.fvg.it (J.P.Z.); zilli.mauriziomd@gmail.com (M.Z.)

**Keywords:** celiac disease, intestinal anti-endomysium antibodies, pediatric patients, adult patients

## Abstract

Intestinal anti-endomysium antibodies are a specific marker of celiac disease. The diagnostic accuracy of this marker seems high in pediatric patients and has not yet been investigated in adults, so the aim of this prospective multicentric study was to evaluate the specificity and sensitivity of this marker in childhood and adulthood. Pediatric and adult patients undergoing intestinal endoscopy for any intestinal condition were enrolled. Serological celiac disease markers and HLA type were evaluated in all patients. Intestinal biopsies were analyzed for standard histology and for intestinal anti-endomysium antibodies with biopsy culture assay. In this study, 291 patients (145 adults and 146 children) were included. In the adult population, 34 were diagnosed with celiac disease, 105 were controls, and, in 6, celiac disease was not confirmed. In the pediatric population, 77 were diagnosed with celiac disease, 57 were controls, and, in 12, celiac disease was not confirmed. High diagnostic sensitivity and specificity of intestinal anti-endomysium antibodies were confirmed in children and additionally proven in adults. To conclude, we can affirm that intestinal anti-endomysium antibodies can be detected with high diagnostic accuracy in both children and adults. The implementation of this marker in the diagnostic work-up would help clinicians to correctly identify celiac disease.

## 1. Introduction

Celiac disease (CD) is a systemic gluten-dependent autoimmune disorder elicited in genetically susceptible subjects that primarily affects the small intestine. The prevalence of around 1% in the general population makes this condition one of the most common lifelong disorders. CD is characterized by wide variability, with patients complaining of intestinal and/or extraintestinal symptoms and patients completely asymptomatic [1,2,3]. Gluten ingestion in celiac subjects induces villous atrophy and a specific immune response characterized by the production of specific autoantibodies: anti-tissue transglutaminase (anti-ttg) and anti-endomysium (AEA) [4,5,6].

The high specificity and sensitivity of these antibodies led to the possibility of CD diagnosis without biopsy. In pediatric patients with anti-ttg values higher than 10 upper normal limit and positive AEA, the non-biopsy approach has a positive predictive value (PPV) > 99%. This means that a group of pediatric CD patients can benefit from the non-biopsy approach, reducing the burden, risks, and costs related to endoscopy procedures [7,8,9,10,11,12]. Recent studies showed that a serology-based CD diagnosis without biopsy could also be reliable in adults with suspicion of CD [13,14,15]. However, the World Gastroenterological Association guidelines recommend a no-biopsy algorithm in adults in countries with limited healthcare resources [16]. 

In symptomatic patients testing positive for serum CD antibodies (anti-ttg and AEA) with intestinal villous atrophy, the diagnosis of classical CD is clear-cut. However, the diagnosis of CD can be challenging when the clinical manifestations are characterized by extraintestinal symptoms not related to intestinal injury. This condition can be diagnosed as potential CD, characterized by serum CD-antibody positivity, usually at low concentration (2–3 times higher than the cut-off), but normal intestinal mucosa, or as seronegative CD with intestinal villous atrophy but negative CD serological markers. In both potential and seronegative CD patients, a gluten-free diet is an effective therapy for improving the clinical condition and stopping the production of disease-specific antibodies [17].

Serum CD antibodies are produced by intestinal B cells and can be detected at the intestinal level in the early phases of the disease when the duodenal mucosa is still normal and serum CD antibodies are negative or positive at a low title. Therefore, intestinal CD antibodies are a specific and sensitive marker of CD to identify patients with potential or seronegative CD [15]. Intestinal CD antibodies can be investigated by using both the double immunofluorescence staining on intestinal cryosections and the anti-endomysium assay on the supernatant of cultured intestinal biopsies (intestinal AEA). Both assays showed high sensitivity and specificity. However, the anti-endomysium assay on biopsy culture supernatant is less demanding and can be performed using commercial kits [15].

In pediatric cases, the diagnostic specificity of intestinal CD antibodies is between 95 and 98%, while the sensitivity is 100% [15]. In adults, nevertheless, it is well known that the reliability of serum antibodies is high, like in pediatric-age patients, and data about the specificity and sensitivity of intestinal CD antibodies are not yet available.

In this prospective and multicentric study, five different gastroenterological units from North–East Italy were involved and both sensitivity and specificity of intestinal AEA were evaluated in pediatric and adult patients suspected of CD or other gastro-intestinal disorders.

## 2. Materials and Methods

### 2.1. Study Design and Patient Population

This was a multicentric study. Adult patients were prospectively recruited in four different gastroenterological units in North–East Italy: Hospital of Cattinara in Trieste, IRCCS National Cancer Institute in Aviano, Hospital of Santa Maria degli Angeli in Pordenone and Hospital of Santa Maria della Misericordia in Udine.

Pediatric patients were prospectively recruited at the Institute for Maternal and Child Health-IRCCS Burlo Garofolo in Trieste. 

All pediatric and adult patients were consecutively recruited from December 2019 to December 2022. We had a delay due to the SARS-CoV-2 pandemic. This study was approved by the hospitals’ independent ethical committee (CEUR-2019-Os-157). Written informed consent was obtained from adult patients or from parents of the children enrolled.

Estimating an intestinal AEA sensitivity and specificity of 90% with an estimation error of 10%, and assuming a prevalence of CD of 25% in adults and 40% in children, the largest sample sizes needed to carry out the study were 139 for adults and 87 for children.

Children and adults who underwent an elective upper gastrointestinal endoscopy for suspicion of CD or other intestinal disorders were included in this study. We excluded patients on a gluten-free diet, those with bleeding disorders, those with no indication for intestinal biopsy in the diagnostic process, and pediatric patients who met the criteria for a no-biopsy and serology-based CD diagnosis [8]. The diagnosis of CD was based on new ESPGHAN (European Society for Pediatric Gastroenterology, Hepatology and Nutrition) guidelines for children, and on NICE (National Institute for Health and Care Excellence) guidelines for adults.

In all enrolled patients, both serum and intestinal CD antibodies were detected, HLA genotype was determined, and small bowel biopsy samples were histologically evaluated.

Symptomatic or asymptomatic pediatric and adult patients were eventually classified as follows: 

Classical CD: patients with positive CD serology (anti-ttg and AEA) and villous atrophy (Marsh classification 2 or 3);

Potential CD: patients with positive CD serology, but normal intestinal mucosa (Marsh classification 0 or 1);

Seronegative CD: patients with negative serology and villous atrophy (Marsh classification 2 or 3);

Controls: patients with negative CD serology who received a diagnosis of a gastro-intestinal disease not CD-related (e.g., gastritis, duodenitis, eosinophilic esophagitis, inflammatory bowel disease, etc.).

### 2.2. Serology Tests

Serum IgA anti-tissue transglutaminase and anti-endomysium were tested in all subjects, including control subjects. Serum IgA anti-ttg was measured using an ELISA assay (Eurospital, Trieste, Italy) following the manufacturer’s instructions, and values higher than 9 U/mL were considered positive. Serum IgA AEA was measured by an indirect immunofluorescence method using a section of monkey esophagus as substrate, as previously described [15]. 

Moreover, IgG anti-ttg and IgG1 AEA were tested in sera of patients with selective IgA deficiency (total IgA < 5 mg/dL).

### 2.3. HLA Type Determination

All subjects were tested for the HLA gene of CD susceptibility using a polymerase chain reaction with allele-specific primers which identify HLA DQ2 and DQ8 (Eurospital Eu-Gene_Risk kit, Trieste, Italy).

### 2.4. Small Bowel Histology

All subjects underwent upper gastrointestinal endoscopy with multiple biopsies: in each patient, two samples were taken from the bulb and two samples from the distal duodenum. The histological analysis was based on Marsh Oberhuber’s Corazza Villanacci Classification. 

### 2.5. Biopsy Culture Anti Endomysium Assay

Two intestinal fragments, one from the bulb and one from the distal duodenum, were cultured for 72 h at 37 °C following the manufacturer’s instructions (Eurospital kit Antiendomysium biopsy, Trieste, Italy). 

Intestinal IgA AEA secreted into culture supernatants were detected in undiluted supernatants, as previously described [18]. Intestinal IgM AEA was investigated in patients with selective IgA deficiency.

### 2.6. Statistics

Data are reported as median ± standard deviation for continuous variables and as proportions for categorical variables. 

The area under the curve of the receiver operating characteristic curve was calculated in order to measure sensitivity and specificity (with a 95% confidence interval [CI] of anti-ttg concentrations as a diagnostic tool to predict atrophic intestinal mucosa). An area under the curve of >0.90, from 0.70 to 0.90, and from 0.50 to 0.70 indicate high, moderate, and low accuracy, respectively. Moreover, the optimal cut-off of anti-ttg concentrations was measured by using the receiver operating characteristic curve analysis, maximizing the simultaneous sum of sensitivity and specificity (Youden index). A value of *p* < 0.05 was considered significant.

Statistical analysis was carried out with the software GraphPad Prism version 4.0.

## 3. Results

**Figure 1:**
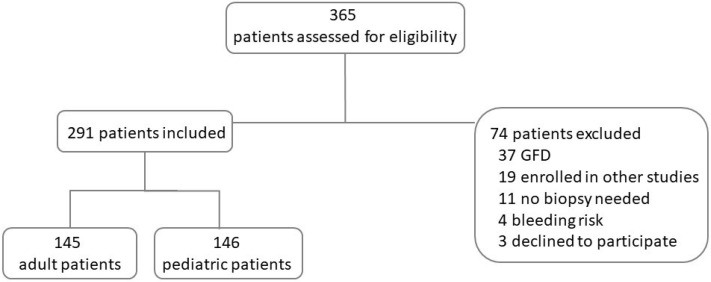
Flowchart of patients assessed, included, and excluded. GFD, gluten-free diet.

Between December 2019 and December 2022, 365 patients were referred for an elective upper gastrointestinal endoscopy to one of the units involved in this multicentric study. Following our eligibility criteria, a total of 291 patients were included in our study: 145 adults (median age 46 years, range 18–84) and 146 children (median age 11 years, range 1–17) (Figure 1).

### 3.1. Adult Subjects

**Figure 2:**
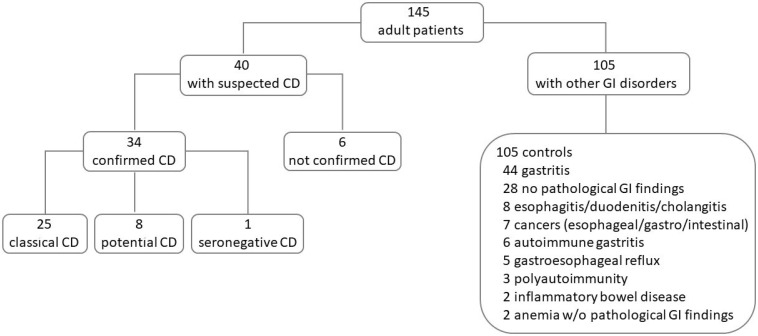
Adult patients’ flowchart. CD, celiac disease; GI, gastro-intestinal.

Forty adult patients out of the 145 included were enrolled for clinical and/or serological suspicion of CD. One hundred and five out of 145 had other gastro-intestinal disorders and were enrolled as a control group (Figure 2). The demographics and disease characteristics of patients included in this study are reported in Table 1.

Among the 40 suspected CD patients, the diagnosis, based on the histological and serological data, was confirmed in 34/40 (85%) and excluded in 6/40 (15%). All 34 confirmed CD patients had the genetic predisposition (HLA DQ2/8) and were diagnosed as follows:

Classical CD patients—twenty-five/34 (12 females and 13 males; median age 29.5 years, range 20–48) were symptomatic patients (Table 1), had a serology with both anti-ttg (170 ± 60 U/mL) and AEA positivity, had intestinal atrophy (Marsh 3); intestinal AEA was found in all 25 classical CD patients with a 100% sensitivity. Three/25(12%) had other autoimmune-associated disorders (Table 1).

Potential CD patients—eight/34 (8 females; median age 45 years, range 18–71) were symptomatic patients (Table 1), had positive serology for anti-ttg (24 ± 37 U/mL) and/or AEA, had normal intestinal mucosa (Marsh 0/1); intestinal AEA was found in all eight potential CD patients. 

Seronegative CD patients—one of 34 confirmed CD (1 female; age 69 years) patients had intestinal symptoms (Table 1), negative serology for both anti-ttg (2 U/mL) and AEA, intestinal atrophy (Marsh 3), and positivity for intestinal AEA. 

Six out of 40 suspected CD patients (six females; median age 44 years, range 36–56), in which CD was excluded, had the genetic predisposition, negative serology for anti-ttg (3 ± 2 U/mL) and AEA, normal intestinal mucosa (Marsh 0), and negative intestinal AEA. Five/6 (83%) were symptomatic patients, 1/6 (17%) had other autoimmune-associated disorders, and 1/6 (17%) showed IgA deficiency (Table 1).

In the control group, 105 symptomatic patients (69 females and 36 males, median age 55 years, range 19–85) suffering from other gastro-intestinal disorders were included (Table 1 and Figure 2). Five/105 (5%) had a family history of CD, 6/105 (6%) had other autoimmune-associated disorders, and 1/105 (1%) showed IgA deficiency (Table 1). The HLA DQ2/8 was positive in 80/105 (76%), serum anti-ttg (3 ± 1.7 U/mL) and AEA were negative in all 105, and intestinal mucosa was normal in 100/105 (95%). Five out of 105 (5%) control patients showed mucosal atrophy: three subjects with Marsh 2 received a diagnosis of sarcoidosis, gastrointestinal non-Hodgkin lymphoma, and gastrointestinal stromal tumor of the stomach, and two subjects with Marsh 3 received a diagnosis of atrophic gastritis. Intestinal AEA was negative in all 105 control patients showing a specificity of 100%. 

The receiver-operating characteristic curve was used to estimate the serum anti-ttg concentration which might predict intestinal atrophy. Anti-ttg serum concentrations above 147 U/mL predicted intestinal atrophy in 14 of 25 classical CD patients (56%) with a sensitivity of 56% (95% CI, 35–76%), a specificity of 100% (95% CI, 69–100%), and positive and negative predictive values of 100% (95% CI, 77–100%) and 42% (95% CI, 77–100%), respectively.

### 3.2. Pediatric Subjects

**Figure 3:**
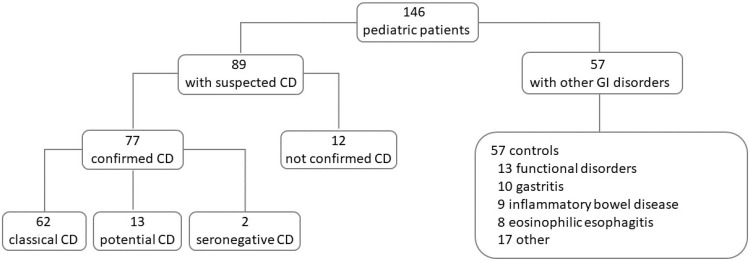
Pediatric patients’ flowchart. CD, celiac disease; GI, gastro-intestinal.

Of the 146 children included in this study, 89 were enrolled for a clinical and/or serological suspicion of CD, and 57 were enrolled for other gastrointestinal disorders as a control group (Figure 3). The demographics and disease characteristics of patients included in this study are reported in Table 2.

The diagnosis of CD was confirmed in 77 of the 89 suspected CD (87%) and excluded in 12/89 (13%). All 77 confirmed CD patients had the genetic predisposition and were diagnosed as follows:

Classical CD patients—sixty-two/77 (37 females and 25 males; median age 9 years, range 2–18) tested positive for anti-ttg (15 ± 51 U/mL) and AEA, showed intestinal atrophy (Marsh 3); intestinal AEA was detected with 100% sensitivity. Fifty-one/62 (82%) were symptomatic patients, 14/62 (6%) had a family history of CD, and 4/62 (6%) had other autoimmune-associated disorders (Table 2).

Potential CD patients—thirteen/77 (10 females and 3 males; median age 10 years, range 13–18) had positive serology for anti-ttg (13 ± 8 U/mL) and AEA, had normal intestinal mucosa (Marsh 0/1); intestinal AEA was detected in 12/13 potential CD with a sensitivity of 92%. Eleven/13 (85%) were symptomatic patients, 2/13 (25%) had a family history of CD, 1/13 (8%) had other autoimmunity (type 1 diabetes), and 1/13 (8%) showed IgA deficiency (Table 2).

Seronegative CD patients—two/77 (2 females of 14 and 18 years) had a negative serology for both anti-ttg (3.5 ± 1 U/mL) and AEA, had intestinal atrophy (Marsh 3); intestinal AEA was detected in both patients. Both were symptomatic and had other autoimmunity (thyroiditis) (Table 2).

Twelve out of 89 suspected CD patients (4 females and 8 males; median age 12 years, range 5–18) in which CD was excluded had the genetic predisposition, negative serology for anti-ttg (3 ± 2 U/mL) and AEA, normal intestinal mucosa (Marsh 0), and negative intestinal AEA. Seven/12 (58%) were symptomatic patients, 7/12 (58%) had a family history of CD, and 2/12 (17%) had autoimmune-associated thyroiditis (Table 2).

In the control group, 57 children (25 females and 32 males, median age 14 years, range 2–18) suffering from other gastrointestinal disorders were included (Table 2, Figure 3). Fifty-three/57 (93%) were symptomatic children, 1/57 (2%) had a family history of CD, and 3/57 (5%) had other autoimmune-associated disorders (Table 2). The HLA DQ2/8 was positive in 28/57 (49%), serum anti-ttg (0.1 ± 0.6 U/mL) and AEA were negative, and intestinal mucosa was normal in all 57 control children. Intestinal AEA was negative in the control group, revealing a 100% specificity. 

## 4. Discussion

In this multicentric study, the biopsy culture AEA assay showed very high sensitivity and specificity in detecting intestinal CD-related autoantibodies and correctly identifying patients with CD both in adulthood and childhood. Its diagnostic accuracy was maintained in classical CD but also in those forms of CD in which conventional serology and histology are inconclusive such as potential and seronegative CD.

Intestinal AEA is currently not included in the diagnostic criteria for the diagnosis of CD, neither in children nor in adults. Nevertheless, it may be a useful diagnostic marker, particularly in atypical CD manifestations that may occur in both pediatric and adult patients. 

In this study population, potential CD was found in both pediatric and adult CD patients with a prevalence of 17% and 23%, respectively. Intestinal AEA was found in all patients with potential CD, suggesting that it may be a useful diagnostic marker when histology is inconclusive.

Another clinical scenario is the case of patients with seronegative villous atrophy in whom, although the number of pediatric and adult patients was small, intestinal AEA proved to be very accurate in separating the patients with CD from the controls.

In addition, checking for the presence of intestinal AEA enables not only the recognition of the different versions of CD but also the exclusion of CD in genetically predisposed subjects with suspected CD who are characterized by negative serology, histologically normal intestinal mucosa, and negative intestinal AEA.

Of interest, patients with IgA deficiency, who represent 2–3% of the CD population [19], were included in this study and tested for intestinal IgM AEA. The diagnostic accuracy of this marker was also retained in these patients.

Intestinal AEA showed a high specificity for excluding CD in control patients with other gastrointestinal disorders. This is in agreement with our previous observations in pediatric populations in which the specificity of intestinal AEA was between 97 and 99% [20]. Absolute specificity of intestinal AEA was also observed in adult control patients suffering from gastrointestinal disorders (e.g., intestinal lymphoma, gastritis, esophagitis) and showing duodenal villous atrophy not CD-related.

Intestinal AEA could be a specific and sensitive marker of CD which could be used to make a prompt diagnosis in symptomatic potential and seronegative patients who may benefit from a well-founded diagnosis reducing both unnecessary medical investigations and delays in diagnosis and treatment [3,21]. One previous observation on potential CD in children showed that the presence of these intestinal antibodies can help to identify those children with potential CD who will develop flat mucosa [22]. Unfortunately, this marker has not been investigated in adults. This study evaluated the reliability of intestinal AEA both in the pediatric and the adult population, and the results confirmed the diagnostic value of this marker which should be implemented in routine diagnostics in children as in adults. 

As a limitation of this study, we acknowledge that the number of patients with potential and seronegative CD in this cohort is small, and further studies, particularly in adults, are needed to truly assess the implementation of this marker in the diagnostic work-up of CD. International guidelines for the diagnosis of CD in adults suggest that the endoscopy procedure be repeated in potential CD patients every 1–2 years. The same guidelines pointed out a delay in the diagnosis of CD in adults that can reach 10 years from the appearance of the first symptoms [16]. Intestinal AEA should be investigated to improve the recognition of this condition and to reduce patient burden, economic costs, and diagnostic delay, especially in adult patients. 

According to our results, in the adult population, serum anti-ttg concentrations higher than 147 U/mL are predictive of intestinal damage. This observation, although the number of patients is small, is in agreement with a recent multicentric study in adults in which serum anti-ttg concentrations greater than 10 times the upper normal limit were predictive of duodenal villous atrophy. This indicates that a serology-based CD diagnosis without biopsy could be possible in adults with suspicion of CD and high serum anti-ttg concentrations, as already recommended by pediatric guidelines. 

## 5. Conclusions

In conclusion, our results confirmed the high diagnostic accuracy of intestinal AEA in children and revealed high sensitivity and specificity in adults, too. The biopsy culture AEA assay should be applied in pediatric and adult gastroenterology to further demonstrate the applicability of intestinal AEA in the diagnostic routine of CD. Finally, the inclusion of this marker in the diagnostic work-up for celiac disease should be considered in both adulthood and childhood.

## Figures and Tables

**Table 1:** Demographic and disease characteristics of adult patients.

	Classical CD n = 25	Potential CD n = 8	Seronegative CD n = 1	Not-Confirmed CD n = 6	Controls n= 105
Female, n (%)	12 (48%)	8 (100%)	1 (100%)	6 (100%)	69 (66%)
Age yr, median (range)	29.5 (20–48)	45 (18–71)	69	44 (36–56)	55 (19–85)
Symptoms, n (%)	25 (100%)	8 (100%)	1 (100%)	5 (83%)	105 (100%)
intestinal	22	8	1	4	104
extraintestinal	4	2	–	1	6
Family history of CD, n (%)	–	–	–	–	5 (5%)
Autoimmunity, n (%)	3 (12%)	–	–	1 (17%)	6 (6%)
diabetes type 1	1	–	–	–	–
gastritis	–	–	–	1	6
thyroiditis	2	–	–	1	3
IgA deficiency, n (%)	–	–	–	1 (17%)	1 (1%)

**Table 2:** Demographic and disease characteristics of pediatric patients.

	Classical CD n = 62	Potential CD n = 13	Seronegative CD n = 2	Not-Confirmed CD n = 12	Controls n= 57
Female, n (%)	37 (60%)	10 (77%)	2 (100%)	4 (33%)	25 (44%)
Age yr, median (range)	9 (2–18)	10 (13–18)	16 (14–18)	12 (5–18)	14 (2–18)
Symptoms, n (%)	51 (82%)	11 (85%)	2 (100%)	7 (58%)	53 (93%)
intestinal	33	9	1	5	47
extraintestinal	31	7	1	5	17
Family history of CD, n (%)	14 (22%)	2 (15%)	–	7 (58%)	1 (2%)
Autoimmunity, n (%)	4 (6%)	1 (8%)	2 (100%)	2 (17%)	3 (5%)
diabetes type 1	1	1	–	–	2
thyroiditis	3	–	2	2	1
IgA deficiency, n (%)	–	1 (8%)	–	–	–

## Data Availability

The data presented in this study are available on request from the corresponding author due to privacy and ethical reasons.

## Abbreviations

AEA	Intestinal anti-endomysium antibodies
Anti-ttg	Anti-tissue transglutaminase
CD	Celiac disease CD
ESPGHAN	European Society for Pediatric Gastroenterology, Hepatology and Nutrition
GI	Gastro-intestinal
HLA	Human Leukocyte Antigens
NICE	National Institute for Health and Care Excellence
PPV	Positive predictive value
